# Assessment of suitable referral, effectiveness and long-term outcomes of standard vs intensive pain management programmes for people with chronic pain

**DOI:** 10.1177/20494637221132451

**Published:** 2022-10-11

**Authors:** Jasmine H. Hearn, Sarah Martin, Melanie Smith

**Affiliations:** 1 The Department of Psychology, 5289 Manchester Metropolitan University, UK; 2 Manchester & Salford Pain Centre, Irving Building, 7047 Salford Royal NHS Foundation Trust, UK

**Keywords:** pain management, distress, disability, pain catastrophizing, physical outcomes

## Abstract

**Background:**

Chronic pain is a leading cause of disability, often requiring multidisciplinary management. 2021 NICE guidance has questioned the quality of the evidence surrounding the efficacy of pain management programmes (PMPs), with only minor benefit demonstrated in psychological and physical outcomes. There is need for further high-quality evidence for the efficacy of PMPs for a range of chronic pain conditions and to identify barriers to successful management of chronic pain.

**Objective:**

This service evaluation utilised routinely collected outcome data of 508 PMP attendees to investigate change in pain- and patient-related outcomes across two distinct PMPs; a standard and an intensive PMP, and establish their longer-term efficacy and appropriateness for patients with differing degrees of need.

**Results:**

More people with chronic widespread pain, fibromyalgia, and osteoarthritis were referred to the intensive PMP (reflecting greater disability and distress in these conditions). Those referred to the intensive PMP demonstrated greater distress (such as more severe depression and anxiety), lower pain acceptance and poorer physical function. Improvements were observed in all outcomes across both PMPs (including physical function, pain catastrophising and pain acceptance). Depression and disability demonstrated clinically meaningful improvements in the intensive PMP, and pain severity showed clinically meaningful improvement in both PMPs. However, depression severity, disability, pain severity, and pain interference significantly deteriorated at 6-month follow-up for those on the intensive PMP, with pain severity increasing to a clinically meaningful degree (by more than 10%), though these outcomes remained better than at baseline.

**Conclusion:**

This evaluation identified that people with chronic pain most at risk of deterioration in physical and psychological wellbeing after completing a PMP require early identification to mitigate such deterioration. Established and emerging PMPs need to be tailored to the needs of this group, particularly at follow-up to reduce risks of pain severity increasing, alongside establishing/reinforcing safeguards against deterioration post-PMP.

## Introduction

Pain is a global healthcare challenge, associated with long-term disability and reduced quality of life.^1^ Chronic back and neck pain are some of the leading causes of years lived with disability internationally, along with other chronic pain conditions also featuring in the top 10 causes of disability.^2^ In the UK, estimates suggest that between 13 and 50% of adults live with chronic pain, with 10.4–14.3% reporting moderate to severe disabling chronic pain.^3^ Because of this, it is important to understand the complex interplay of factors that influence pain and pain outcomes from a biological, psychological and social perspective. Likewise, treatment/management interventions need to consider these factors to optimize outcomes for individuals living with chronic pain.

In order to address the complex impacts of chronic pain and the needs of individuals living with it, comprehensive treatment/management is required, covering medical management, activity and physical rehabilitation, and psychological and social elements. Pain management programmes (PMPs), rehabilitative programmes underpinned by psychological principles and self-management training, are considered the gold-standard of intervention for people whose quality of life is affected by chronic pain.^4^ Usually delivered in small groups to enhance social support and shared learning and experience, these multidisciplinary programmes incorporate cognitive-behavioural principles with physical rehabilitation, with the aim of improving physical and psychological functioning (i.e. reducing disability and improving physical movement, and symptoms of depression and anxiety).^4^

Pain management programmes do not focus specifically on pain reduction, instead aiming to address attitudes and behaviours that influence quality of life (such as fear of movement), and improve self-management and independence, helping participants live as normally as possible within any constraints of their pain problem. To achieve this, PMPs contain guided practice on exercise and activity management, goal setting, identifying and changing unhelpful beliefs and ways of thinking, relaxation and changing habits which contribute to disability.^4^ There is good evidence for the efficacy of both outpatient and inpatient cognitive-behavioural interventions, compared with either no treatment or treatment as usual, with greater gains achieved with programmes of greater length and intensity.^4^ Indeed, whilst both in-patient/residential and out-patient/longer programmes demonstrate benefit, more intensive interventions result in greater and more sustained gain.^5^ Consistently, evidence demonstrates that psychological and physical functioning are much improved after completing a PMP, and systematic review evidence recommends further focus on outcomes and the exploration of the different treatment components to establish ‘active ingredients’ for PMPs and for which sub-groups those components work best.^6^

In contrast, however, 2021 NICE guidance^7^ has called into question the quality of the evidence surrounding the efficacy of PMPs for chronic low back pain, finding that most of the evidence reviewed showed no difference in quality of life, nor other outcomes, for people with chronic back pain attending PMPs compared with usual care or waiting list controls. This resulted in the NICE committee being unable to make a recommendation for or against the use of pain management programmes for chronic back pain. Where the population referred to people with mixed pain, the NICE guidelines found evidence for PMPs initiating only minor benefit for physical function and psychological distress. Likewise, the NICE committee noted that most of the evidence for quality of life was from people with chronic low back pain, with the exception of a single small-scale study in people with knee pain.

The new guidance has received substantial criticism, being described as an oversimplified approach to a complex issue.^8^ For example, the guidance separates psychological intervention from physical intervention, despite these being used in conjunction in PMPs, and presents an oversimplified definition of pain itself and a ‘one-size-fits-all’ approach, which undermines the unique impacts and treatment demands of individual pain conditions.^9^ The Faculty of Pain Medicine^10^ has expressed concerns that the guidance poses a risk to the commissioning of PMPs, along with the potential withdrawal of useful treatments and interventions (including PMPs and medications such as non-steroidal anti-inflammatory drugs) from patients in need of these very interventions. The British Pain Society^11^ have also commented on the risk of marginalisation and stigma of those experiencing chronic pain is increased through the recommendation of ‘antidepressants’, and the fostering of patient passivity by excluding interventions that empower patients to live well with pain from the recommendations. This evaluation, therefore, aims to provide further support for the utilisation and effectiveness of PMPs in clinical practice, and for a range of chronic pain conditions, as well as to identify any potential factors that may act as barriers to successful management of chronic pain.

The Manchester and Salford Pain Centre (MSPC) takes a dual approach to the delivery of PMPs for people with chronic pain, providing both a ‘standard’ and an ‘intensive’ PMP. Patient outcome data demonstrates improvements across a range of standardised measures which are taken as per the guidance from The British Pain Society, with those attending a PMP demonstrating improvements in interpersonal relationships, mood and anxiety, increased physical fitness, increased likelihood of return to work, optimised medication use and reduced health care use. As part of routine service evaluation, MSPC collects demographic data, and data on physical and psychological function of those attending the PMPs delivered. As such, MSPC has maintained a large record of outcome data that offers the opportunity for in-depth analysis of over 500 PMP attendee’s data, much of which reflects a variety of demographics, such as gender and employment status, and pain conditions such as back pain, neurological (e.g. neuropathic) pain, chronic widespread pain and fibromyalgia. Analysis of such a dataset can enable greater confidence in drawing conclusions about the efficacy of PMPs across a range of pain conditions, and across two distinct PMP delivery modes (intense vs standard) to ensure that the appropriate PMP is being recommended for those who present with chronic pain.

The purpose of the present service evaluation, therefore, was to utilise routinely collected outcome data from the MSPC to investigate the differences in pain- and patient-related outcomes across the two distinct PMPs delivered at the clinic. Novel to the evidence base, this evaluation aimed to:

(1) Compare baseline clinical & demographic metrics for each PMP type to establish whether the initial screening process is identifying those in greater distress, and ensuring that they are then being appropriately referred for more intensive treatment, and

(2) explore the efficacy of each PMP individually, including longer-term follow-up to 6-months post-PMP, with reference to any clinically meaningful changes in addition to statistical change.

## Materials and methods

### The programmes

A PMP is a rehabilitative programme based upon evidence-based, cognitive-behavioural and acceptance and commitment principles for people whose lives are adversely affected by chronic pain. The overall aim of a PMP is to reduce the disability and distress caused by chronic pain, by teaching people living with pain various physical, psychological and practical techniques to improve quality of life. Pain management programmes are carried out in a group format (usually 8 to 12 patients) to maximise possibilities of learning from others. The British Pain Society advocate that a PMP requires input from a medically qualified person, a Health and Care Professions Council (HCPC) registered physiotherapist with experience in managing people with chronic pain and a chartered (HCPC) clinical psychologist, or other registered (HCPC) practitioner psychologist with appropriate pain training. The psychologist and the physiotherapist on the PMP deliver 90% of the content given the focus on rehabilitation with a consultant anesthetist providing support on pain education and medications.

**Figure 1.**
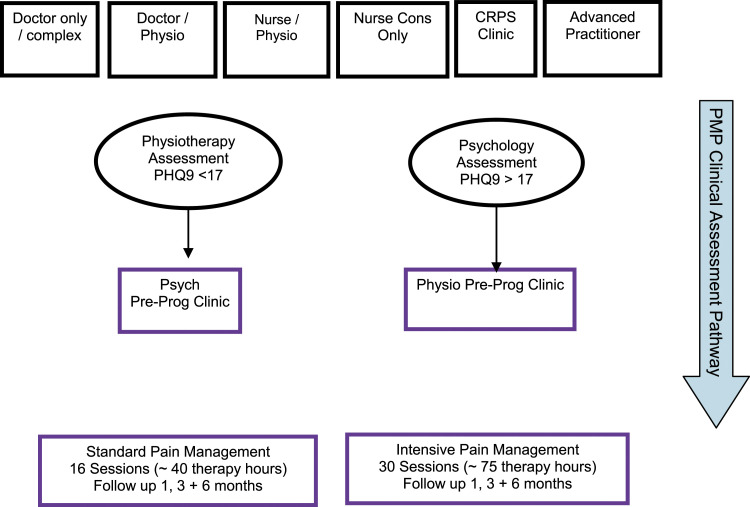
Patient screening and referral process.

Pain management programmes have been delivered at Manchester and Salford Pain Centre since the 1980s. At this time, the PMP was similar in therapy hours to the current intensive PMP but as the centre grew, it was recognized that a different population of service users better orientated to rehabilitation would benefit from a lower ‘dose’ of input. Furthermore, these potential participants were also more likely to be working and therefore logistically, fewer therapy hours would be more accessible for them. This led to the development of the standard PMP which began at the centre in 2002. Both programmes have continued since then, with PMP clinicians assessing potential participants on a standardised rehabilitation pathway (see Figure 1 and ‘patient screening and referral’). In 2013, the British Pain Society highlighted the need for PMPs to cater for variation in levels of disability and distress within the chronic pain population and recommended that service users were able to access different levels of intensity for therapeutic input most suited to patient need,^4^ which aligns with the delivery of PMPs at the pain centre.

### The intensive programme

The intensive programme runs Monday to Friday, 9.30a.m. to 4.30p.m., over 3 consecutive weeks (75 therapy hours). Patients attending this programme typically have higher levels of pain related distress, fear and disability that has been identified through the assessment pathway.

### The standard programme

The standard programme runs for 2 days per week, 9.30a.m. to 4.30p.m., over 4 consecutive weeks (40 therapy hours). Patients attending this programme demonstrate lower levels of distress and disability.

Table 1 lists examples of the interventions used across the PMPs. Both PMPs utilise all interventions listed, though the intensive programme dedicates around double the time to each intervention. The standard programme uses between session tasks for participants to apply concepts learned in sessions to their situation and bring to next session to discuss whereas in the intensive PMP, patients are supported by staff to apply educational components and key concepts to themselves. For both PMPs, there are 2 half-day follow-up sessions at intervals of 1 month and 3 months after the programme. Six months after attending the PMP, patients attend an individual appointment for follow-up assessment to examine physical and psychological outcomes.

**Table 1.** List of interventions/strategies utilised in both PMPs.

Interventions/strategies used in PMPs	
Medic led	• Education about chronic pain neurophysiology and validation of pain management rehabilitation approach
	• Medication review and advice
	• Education about chronic pain medications and opportunity for Q&A
	• All patients have 1:1 medication review with the medic towards the end of the PMP
Physiotherapy led	• Physical reconditioning – Daily graded whole body stretching, strengthening and aerobic exercise programme
	• Activity management - pacing and pacing up activity^a^
Psychology led	• A cognitive behavioural approach to pain and stress^a^ **–** education around rationale of a biopsychosocial approach to stress in chronic pain and cognitive behavioural strategies to address behaviours that maintain maladaptive coping
	• Cognitive behavioural approach to problem solving training^a^ **–** education and working through 5 stages of problem solving for life areas negatively impacted by chronic pain
	• Applied relaxation training – Experiential diaphragmatic breathing, progressive muscle relaxation and autogenic relaxation
	• Cognitive behavioural sleep education and management – Psychoeducation about sleep and cognitive behavioural maintaining factors. Cognitive behavioural strategies to manage insomnia
Joint psychology and physiotherapy sessions	• Goal planning^a^ – turning problem solving areas into SMART goals
	• Flare-up management – Education about flare-ups and creation of flare-up plan
	• Partner/friend/family member session – information session and Q&A opportunity for family member/friend chosen by PMP participant and big group discussions around case studies^a^
	• Chronic pain and intimate relationships^a^ **–** participants use CBT skills to formulate cognitive behavioural factors contributing to impact of pain on couple’s relationship and problem solve solutions as a group
	• Maintaining change – Use of prochaska and di- clemente model of behaviour change to anticipate barrier to application of PMP skills using group discussion and worksheets

^a^Session(s) introduce key concepts, hypothetical case study of person with chronic pain to highlight concepts, large/small group discussion of case study to highlight application of concepts to person, patient applies to own situation.

### Patient screening and referral

Patients follow a routine pathway of assessment for PMPs (Figure 1). Following attendance at initial clinics focused on providing medical examination (Table 2 presents criteria by which patients are assessed for eligibility for referral to a PMP), they attend either a physiotherapy assessment clinic or psychology assessment clinic contingent on questionnaire scores gathered via the Patient Health Questionnaire (PHQ9) prior to their first appointment. Those scoring less than 17 on this (cut score for moderately-severe depressive symptoms) attend a 60 min PMP physiotherapy assessment, whilst those scoring 17 or more attend a 90 min PMP psychology assessment. All patients have a further 60 min assessment with the alternative practitioner to complete the assessment before signing a commitment and consent form for the PMP.

**Table 2.** Eligibility criteria for referral to a PMP.

Eligibility criteria for referral to a PMP
Chronic pain causing significant disability and/or distress
All appropriate investigations and treatments completed
No planned referrals to other specialities regarding the pain problem
Co-morbid problems should not be risk factors for active rehabilitation (e.g. uncontrolled angina or asthma)
Can manage basic ADL and is self-caring
No major substance misuse (including alcohol)
No major psychiatric issues of current significance
The patient is willing to participate in a group programme involving psychological and activity-based interventions

Both assessments follow a routine biopsychosocial assessment to identify patients who are orientated to rehabilitation and are likely to benefit from a PMP, gathering information about the impact of pain on activity and mood, current coping strategies and collaborating with the patient to identify goals for the PMP. The psychology assessment focuses on pain beliefs and coping, impact of pain on social function and mood, a brief psychosocial history, and the patient’s social and employment circumstances. Given that many patients present with clinical levels of depression at referral to the centre, a key aim of the psychology assessment is to establish the degree to which factors contributing to depression are pain related and therefore manageable on a PMP or related to other psychosocial stressors and therefore require primary care input. If both are present, the psychology assessment aims to establish whether pain interventions or primary mental health interventions would best meet the person’s needs. The physiotherapy assessment explores pain beliefs, coping and activity, in addition to checking that the patient’s current health status and physical function is sufficient for them to participate in a group rehabilitation format. If a PMP is not appropriate for their needs, an alternative rehab format is identified. Clinicians use these clinical assessments to decide which PMP is most suitable for the patient’s needs. Clinicians and patients collaboratively identify 4–6 goals for the PMP based on their presentation that often incorporate physical and psychological change the patient wishes to achieve through the PMP.

### Outcomes

Since September 2014, both physical and psychological outcomes have been assessed at baseline (pre-PMP), upon completion of the PMP (post-PMP) and at 6-months post-PMP. Measures include:

• **Demographic information:** this includes patient sex, employment status and pain type.
• **Physical measures:** For all of the below measures, patients are asked to complete them without the use of walking aids, if able, and are able to take breaks to rest and sit down as needed.
   ○ *The five-minute walk* is a timed test of the distance, in meters, that a patient can walk within 5 min.
   ○ *The 20-meter walk* is a timed test of the length of time a patient takes to walk the distance of 20 m in seconds.
   ○ *The one-minute step up* is a timed test of the number of times a patient can step up onto a standard exercise step and down again, in 1 minute.
• **Brief Pain Inventory (short-form; BPI):**^12^ The BPI is a 12 item self-administered questionnaire that captures information on pain intensity and interference; Two outcomes were calculated from the BPI: (1) Pain Severity – 5 item subscale comprising average score of least, worst, average and current pain rating on a 10 point likert scale, and (2) Pain Interference – 7 item subscale comprising average of how much pain has interfered with seven daily activities (general activity, walking, mood, enjoyment of life, normal work, relations with others and sleep) on a 10 point likert scale. The BPI has been shown to be an appropriate measure for a broad range of pain conditions.^12^ When considering the clinical significance of changes in pain severity, the IMMPACT study^13^ consider reductions of ≥30% to reflect moderate clinically important differences and reductions of ≥50% to reflect substantial improvements. For the interference subscale, clinically meaningful change is reflected in a 1 to 3 point reduction in mean interference score^14^ depending on pain condition and treatment.
• **Roland and Morris Disability Questionnaire (RMDQ):**^15^ The RMDQ is a self-administered questionnaire which assesses perceived level of physical disability. Greater disability is reflected by higher scores out of a total of 24. Startford and Riddle^16^ suggest a score of 4/24 distinguishes between functional and dysfunctional states. For patients with lower back pain, a clinically meaningful change of a 30% reduction in score between pre and post treatment has been suggested.^17^
• **Patient Health Questionnaire – 9 (PHQ9):**^18^ The PHQ-9 is the self-administered 9-item depression scale of the Patient Health Questionnaire and can be used for a chronic pain population without modification.^19^ Scores of 5, 10, 15 and 20 on the PHQ-9 represent thresholds of mild, moderate, moderately severe and severe depressive symptoms, respectively. A 5-point change is considered to be clinically significant.^20^ Although 10 has been the conventional PHQ-9 cut-off score, a higher score (11 or 12) may be preferable in certain settings.^21^
• **Generalised Anxiety Disorder – 7 (GAD7):** The GAD-7 is a self-administered 7-item questionnaire measuring anxiety symptom presence and severity. As with the PHQ-9, a score of 10 or greater on the GAD-7 represents a reasonable cut point for identifying cases of Generalised Anxiety. Cut points of 5, 10 and 15 are interpreted as representing mild, moderate and severe levels of anxiety on the GAD-7.^22^ Although not a pain measure, the GAD-7 is frequently used in primary care practice and has shown validity and reliability as a measure of anxiety in the general population.^23^
• **Tampa Scale of Kinesiophobia (TSK):** The TSK measures fear of movement and re-injury (kinesiophobia) that is frequently present in patients with chronic pain. It is a 17 item measure with score ranges from 17 to 68 and scores >37 are generally considered as a high level of kinesiophobia.^24^
• **Pain Catastrophising Scale (PCS):** The PCS is a 13-item self-administered questionnaire which provides a total score for pain catastrophising and measures rumination, magnification and helplessness. A higher score is indicative of a higher tendency to catastrophise about pain. Previous studies have shown a cut-off of more than 30 points to be associated with clinical relevance.^25^
• **Chronic Pain Acceptance Questionnaire (CPAQ):** The CPAQ measure patients’ engagement in important daily activities regardless of pain (11 item Activity Engagement Subscale), and relative absence of attempts to control or avoid pain (9 item Pain Willingness Subscale). No clinical cut-off has been identified for this measure although higher scores reflect higher levels of acceptance and willingness to engage in activity despite pain.^26^ The Activity Engagement Subscale alone was used in the PMPs as previous unpublished MSPC analysis indicated that pre-treatment scores on this subscale significantly predicted main PMP outcomes of mood and physical functioning measures.
• **Negative Problem Orientation Questionnaire (NPOQ):** The NPOQ is a 12-item scale that measures the degree to which problems are viewed as a threat to oneself, a propensity to doubt one’s problem-solving ability, and to be pessimistic about outcome. It is primarily a research tool so no cutoffs for a clinical population have been identified.

### Data analysis

The statistical analyses were performed in SPSS software (IBM Corp. V.26, USA) and R software (R Foundation for Statistical Computing V3.5.2, Austria). Analyses were only completed on individuals who completed the PMP. Data were assessed for normality via the Shapiro**–**Wilk and all variables except for the 5-minute walk were non-normally distributed. Baseline differences between the standard and intensive PMP in clinical and demographic metrics was assessed via a Mann**–**Whitney U tests. Analyses of longitudinal changes were assessed via linear mixed model (LMM) repeated measures ANOVA (RM-ANOVA) using the ‘lme4’^27^ and ‘lmerTest’^28^ toolboxes.

The standard and intensive PMP were assessed separately. Linear-mixed models can effectively handle the hierarchal nature of the data and can account for incomplete datasets. In comparison to standard RM-ANOVA or non-parametric equivalent (i.e. Friedman’s), the LMM accounts for missing data via maximum likelihood estimation rather than case-wise deletion, therefore minimizing data loss. Random intercept models were designed such that each participant had an individual slope and intercept, to produce correlation between slope and intercept. Baseline age, gender, diagnosis type and timepoint were included as fixed effects. Each physical and psychological metric was assessed individually for the effect of timepoint. Model fit was assessed via ANOVA using Satterthwaite’s method, plus post-hoc comparison adjusted for Bonferroni correction between each timepoint.

### Statement of ethics

At their final assessment appointment prior to starting the PMP, patients complete and sign a consent form for the PMP. This includes reading through a commitment form highlighting the importance of attending all sessions given that PMP content for both physiotherapy and psychology sessions builds on previous sessions, participating in activities and attending follow-ups. When patients are unable to commit to full attendance on a specific PMP, they are listed for the next available PMP that they are able to attend or offered an alternative intervention format (e.g. individual interdisciplinary sessions).

The consent form outlines the physical and psychological content of the PMP and the aims of the PMP in reducing pain related disability and distress, rather than reducing pain itself. The commitment and consent forms are discussed with each patient prior to signing. This is then revisited at a joint appointment by the clinicians facilitating the PMP immediately before the programme commences. Patients have the right to withdraw at any time from the PMP assessment and intervention pathway without their care being compromised and are offered and alternative format of pain management intervention regardless of where they are in the pathway.

Participants in the PMPs provide consent for their data to be collected for service evaluation and outcome measurement purposes. Through the course of both PMPs, attendees are provided with ongoing signposting and support from PMP clinicians and psychologists. No personal data are included in the dataset, such that the dataset is completely anonymised and contains only patient identifiers that can only be used by PMP clinicians to link follow-up data to previous data points. This service evaluation was reviewed and approved by Northern Care Alliance Research & Innovation (reference number S21HIP31), which was also certified by Manchester Metropolitan University (reference number 34215).

## Results

Overall, 327 attended a standard PMP and 271 attended an intensive PMP (598 total). In the standard PMP, 6 (2 female) did not start, 43 (32 female) did not finish and 278 (195 female) completed all sessions of the standard PMP (treatment completion is defined as attended all PMP sessions). In the intensive PMP, 4 (1 female) did not start, 26 (12 female) did not finish and 241 (170 female) completed all sessions of the intensive PMP.

### Baseline assessment

A similar gender split is reported in both PMP-type: standard: 30% male, intensive: 32.5% male. When compared to the standard-PMP, the intensive-PMP included a higher proportion of individuals presenting with Chronic Widespread Pain/Fibromyalgia Syndrome/Osteoarthritis (CWP/FMS/OA; standard: 21.4%, intensive: 36.9%) and in unemployment (standard: 19.6%, intensive: 51.7%). A chi-square test of homogeneity was run to determine if gender, diagnosis type and employment status frequency differed between PMP types. Sample size was sufficient for all groups.^29^ Gender did not show significant differences between PMP type (χ2 (1) = 0.433, *p* = 0.510), and no post-hoc comparisons were completed. The frequency of diagnosis type and employment status were significantly different between PMP types (diagnosis type: χ2 (8) = 27.433, *p* = 0.001 and employment status: χ2 (3) = 68.120, *p* = 0.000). Observed percentages of diagnosis type and employment status for each PMP type are reported in Table 3. Post-hoc analyses were carried out separately for diagnosis type and employment status. This involved pairwise comparisons using multiple z-tests of two proportions with a Bonferroni correction. Statistical significance for diagnosis type was accepted at *p* < 0.00,625 (0.05/8) and highlighted with ^. Statistical significance for employment type was accepted at *p* < 0.0125 (0.05/4) and highlighted with ^♦^. Compared to the standard PMP, the intensive PMP had higher numbers of individuals with CWP/FM/OA and lower cases of neck and arm pain. Unemployment was more prevalent in the intensive PMP than the standard PMP.

**Table 3.** Baseline demographics for Standard and Intensive PMP.

	Factor	Percentages		Post-hoc chi-square		
		Standard, %	Intensive	Chi-square	df	*p*-value
Gender	Male	30.0	32.5%	—	—	—
	Female	70.0	67.5%	—	—	—
Diagnosis	Back ± hips	15.9	14.0%	0.589	1	0.443
	Back + leg(s)	26.6	29.2%	0.256	1	0.613
	Neck ± shoulder	3.1	2.2%	0.471	1	0.493
	Neck + Arms(s)	5.8	1.5%	**7.850**	**1**	**0.005^**
	Abdo/Pelv/Gynae/Groin	1.5	0.4%	2.090	1	0.148
	CWP/FMS/OA	21.4	36.9%	**15.981**	**1**	**0.000^**
	Local/Neuro/CRPS	6.4	3.3%	3.231	1	0.072
	Other	15.0	11.4%	1.922	1	0.166
	Data missing	1.8	1.1%	n/a	n/a	n/a
Employment	In paid employment	70.9	41.0%	**57.542**	**1**	**0.000^♦^**
	Unemployed	19.6	51.7%	**66.280**	**1**	**0.000*^♦^***
	Retired	7.6	7.0%	0.114	1	0.735
	Full time education	0.6	0.4%	0.184	1	0.668
	Data missing	1.2	None	n/a	n/a	n/a

**NB**: Abdo – Abdomen, Pelv – Pelvis, Gynae – Gynecological, CWP – Chronic Widespread Pain, FMS – Fibromyalgia Syndrome, OA –Osteoarthritis, Neuro – Neurological, CRPS – Chronic Regional Pain Syndrome. Bonferroni correction: Statistical significance for Diagnosis type was accepted at *p* < 0.00,625 (0.05/8) and highlighted with ^. Statistical significance for Employment status was accepted at *p* < 0.0125 (0.05/4) and highlighted with ^♦^. Metrics are reported as percentages.

Baseline comparison for physical and psychological characteristics for both PMP type are reported in Table 4. Baseline age did not differ between PMP type, however, all physical and psychological outcomes were poorer in the intensive-PMP compared to the standard-PMP.

**Table 4.** Baseline age and outcome measure for standard and intensive PMP.

Factor	Standard		Intensive		Man-Whitney U		
	*n*	Median (IQR)	*n*	Median (IQR)	Z	U	*p*-value
Age	327	46 (18)	271	47.00 (15)	1.108	46,638.50	0.268
5 min walk (metres)^	318	290 (111)	268	210 (129)	−10.468	21,246.50	0.000**
20 m walk (seconds)	317	14.8 (6.9)	268	20.10 (11.95)	10.120	63,090.00	0.000**
1 min step (steps)^	317	20 (11)	268	15.00 (9)	−8.615	24,946.00	0.000**
PCS (/52)	313	24 (18)	269	30 (16)	5.751	53,726.50	0.000**
CPAQ (0–66) ^	314	34 (14)	268	26 (14.5)	−7.547	26,937.00	0.000**
PHQ-9 (/27)	314	12 (9)	268	18 (7.75)	8.762	59,773.50	0.000**
GAD-7 (/21)	312	9 (8)	268	13 (7.75)	7.204	56,282.50	0.000**
NPO (/60)	313	24 (15)	269	29 (16.5)	6.304	54,843.00	0.000**
RMDQ (/24)	312	14 (7)	269	18 (5)	10.188	62,469.00	0.000**
TSK-17 (/68)	312	36 (11)	269	39 (2)	3.393	48,806.00	0.001**
BPI severity (/10)	303	5.5 (2)	268	6.25 (1.75)	5.219	50,854.50	0.000**
BPI interference (/10)	295	6 (2.86)	263	7.43 (2.43)	8.803	55,522.50	0.000**

**NB**: PCS – Pain catastrophising scale, CPAQ – chronic pain acceptance questionnaire, PHQ-9 – patient health questionnaire, GAD-7 – generalized anxiety disorder assessment, NPO – negative problem orientation, RMDQ – Roland and Morrise disability questionnaire, TSK-17 – Tampa scale of kenesiophobia, BPI – brief pain inventory. * = *p* < 0.05, ** = *p* < 0.01, *** = *p* < 0.001. A higher score is better in all physical and psychological metrics except for those denoted with ^. For each outcome, the sample size (n), median, and interquartile range (IQR), along with statistical output for the Mann-Whitney U test.

### Longitudinal changes

**Figure 2.**
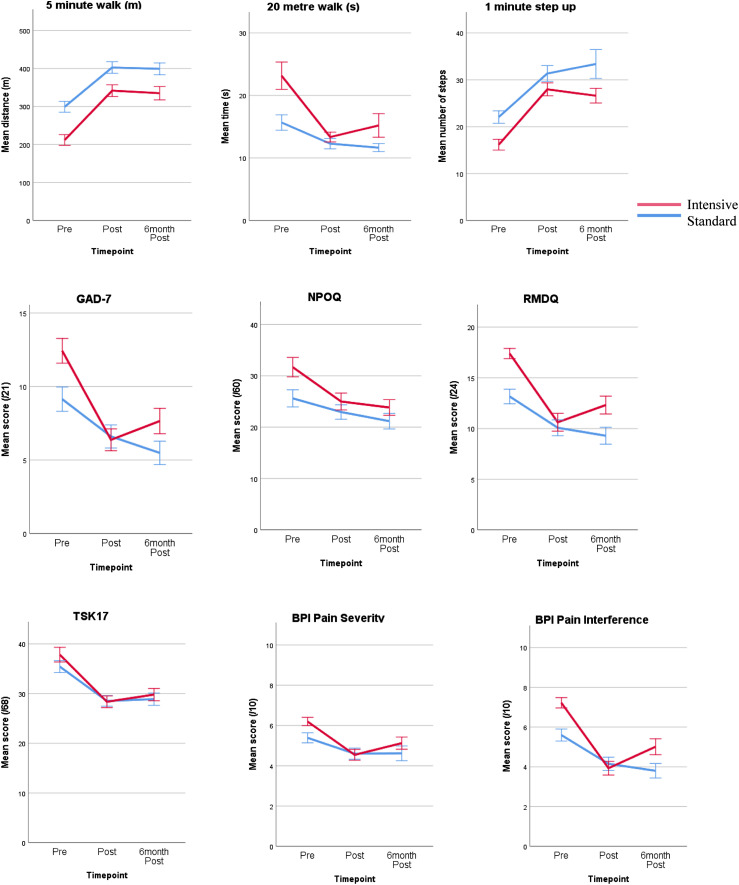
Mean scores across physical and psychological outcomes from pre-PMP, to post-PMP and to 6-month post-PMP.

Table 5 and Figure 2 report the change in each physical and psychological metric for pre-PMP, post-PMP and 6-month post-PMP timepoints. Statistical analyses via LMM RM-ANOVA are reported in Table 6 and all metrics showed an overall significant improvement in both the standard and intensive PMPs.

**Table 5.** Outcome measure scores over time for standard and intensive PMP.

Outcome measure	Standard-PMP			Intensive-PMP		
	Mean (SE)			Mean (SE)		
	Pre	Post	6 month post	Pre	Post	6 month post
5-min walk (metres)^	295 (5.44)	393 (5.59)	394 (6.48)	208 (5.63)	331 (5.82)	329 (6.85)
20 m walk (seconds)	17.9 (1.26)	14.4 (1.27)	13.8 (1.29)	24.8 (0.86)	15 (0.90)	16.2 (1.11)
1 min step (steps)^	21.5 (0.69)	30.8 (0.73)	33.2 (0.95)	15 (0.50)	26.4 (0.52)	25.5 (0.63)
PCS (/52)	23.8 (0.7)	15.2 (0.72)	12.8 (0.81)	29.7 (0.74)	15.7 (0.77)	16.5 (0.87)
CPAQ (0–66) ^	33.0 (0.68)	41.9 (0.70)	44.6 (0.80)	25.5 (0.73)	40.2 (0.77)	39.5 (0.87)
PHQ-9 (/27)	12.37 (0.36)	8.82 (0.37)	7.89 (0.43)	17.1 (0.37)	9.1 (0.39)	11.4 (0.43)
GAD-7 (/21)	9.18 (0.32)	6.55 (0.33)	5.52 (0.38)	12.63 (0.33)	6.62 (0.35)	8.01 (0.39)
NPO (/60)	25.6 (0.60)	23.0 (0.6)	21.1 (0.70)	31.4 (0.69)	25.1 (0.26)	23.9 (0.80)
RMDQ (/24)	13.53 (0.31)	10.4 (0.32)	9.63 (0.37)	17.9 (0.31)	11.4 (0.72)	13.0 (0.37)
TSK-17 (/68)	36.2 (0.49)	29.4 (0.51)	29.6 (0.59)	39 (0.52)	29.3 (0.33)	30.9 (0.62)
BPI severity (/10)	5.55 (0.12)	4.68 (0.13)	4.71 (0.15)	6.18 (0.10)	4.62 (0.55)	5.2 (0.12)
BPI interference (/10)	5.74 (0.13)	4.25 (0.14)	3.95 (0.16)	7.3 (0.13)	4.07 (0.14)	5.17 (0.16)

**NB**: PMP – Pain management programme, PCS – Pain catastrophising scale, CPAQ – chronic pain acceptance questionnaire, PHQ-9 – patient health questionnaire, GAD-7 – generalized anxiety disorder assessment, NPO – negative problem orientation, RMDQ – Roland and Morrise disability questionnaire, TSK-17 – Tampa scale of kenesiophobia, BPI – brief pain inventory. A higher score is better in all physical and psychological metrics except for those denoted with ^. Data presented as mean and standard error (SE).

**Table 6.** Summary of linear mixed model **–** repeated measures ANOVA output statistics for all outcome measures.

Outcome measure	Linear mixed-effects model - RM-ANOVA							
	Standard				Intensive			
	DF	F-value	*p*-value	ηp^2^	DF	F-value	*p*-value	ηp^2^
5 min walk (metres)^	(2, 420.16)	388.3699	<0.001	0.65▲	(2, 398.87)	398.5424	<0.001	0.67▲
20 m walk (seconds)	(2, 394.54)	82.2642	<0.001	0.29▲	(2, 371.5)	70.5755	<0.001	0.28▲
1 min step (steps)^	(2, 457.07)	117.7746	<0.001	0.34▲	(2, 410.16)	332.276	<0.001	0.62▲
PCS (/52)	(2, 471.11)	154.1806	<0.001	0.40▲	(2, 444.43)	197.6919	<0.001	0.47▲
CPAQ (0–66) ^	(2, 478.74)	159.9301	<0.001	0.40▲	(2, 446.88)	216.1227	<0.001	0.49▲
PHQ-9 (/27)	(2, 486.94)	81.8115	<0.001	0.25▲	(2, 447.05)	232.3672	<0.001	0.51▲
GAD-7 (/21)	(2, 480.47)	63.304	<0.001	0.21▲	(2, 445.12)	168.7557	<0.001	0.43▲
NPO (/60)	(2, 468.08)	30.8301	<0.001	0.12◊	(2, 434.03)	72.5545	<0.001	0.25▲
RMDQ (/24)	(2, 480.25)	94.2008	<0.001	0.28▲	(2, 453.1)	214.9023	<0.001	0.49▲
TSK-17 (/68)	(2, 483.08)	124.8244	<0.001	0.34▲	(2, 446.36)	173.4351	<0.001	0.44▲
BPI severity (/10)	(2, 482.31)	30.0432	<0.001	0.11◊	(2, 438.51)	114.1235	<0.001	0.34▲
BPI interference (/10)	(2, 475.22)	82.57	<0.001	0.26▲	(2, 475.22)	82.57	<0.001	0.26▲

**NB**: NB: PMP – Pain management programme, PCS – Pain catastrophising scale, CPAQ – chronic pain acceptance questionnaire, PHQ-9 – patient health questionnaire, GAD-7 – generalized anxiety disorder assessment, NPO – negative problem orientation, RMDQ – Roland and Morrise disability questionnaire, TSK-17 – Tampa scale of kenesiophobia, BPI – brief pain inventory. * = *p* < 0.05, ** = *p* < 0.01, *** = *p* < 0.001. A higher score is better in all physical and psychological metrics except for those denoted with ^. ηp2 effect size: 0.01: ⸋small effect size, 0.06 ◊medium effect size, ▲0.14 or higher: large effect size. Effect sizes interpreted according to Coolican.^32^ Data are presented for Standard and Intensive PMP.

Post-hoc analyses to compare differences between timepoint are presented in Table 7 and Table 8. Post-hoc tests were completed for (1) Pre-PMP vs Post-PMP, (2) Pre-PMP vs 6-month post-PMP and (3) Post-PMP vs 6-month post-PMP. For each timepoint comparison, a statement of whether the scores improved (impr), worsened (worse) or maintained (maint) are included in the tables. Our results demonstrate that both PMP types improve all physical and psychological metrics overall (Pre-PMP vs Post-PMP). The post-hoc tests between post-PMP and 6-month post-PMP report contrasting results for PMP types **–** the standard PMP maintained or improved scores, whilst scores in the intensive PMP maintained or worsened. We comment on the clinical relevance of the change in scores. PCS, PHQ-9, GAD7 and RMDQ scores can be classified into severity levels. We report below whether the significant changes in score resulted in a change in classification.

**Table 7.** Bonferroni corrected post-hoc comparisons between each PMP visit for Standard PMP. The direction of the significant change is noted as improved (Impr), maintained (Maint) or worsened (Worse).

	Post hoc – Standard-PMP											
Outcome measure	Pre vs post				Pre vs 6 month post				Post vs 6 month post			
	DF	T-value	*p*-value	Effect	DF	T-value	*p*-value	Effect	DF	T-value	*p*-value	Effect
5 min Walk^	416	−25.667	<0.0001	Impr	429	−19.664	<0.0001	Impr	422	−0.13	0.897	Maint	
20 m Walk	404	11.246	<0.0001	Impr	405	9.962	<0.0001	Impr	404	1.441	0.150	Maint	
1 min Step-up^	428	−12.923	<0.0001	Impr	469	−12.457	<0.0001	Impr	455	−2.508	0.013	Maint	
PCS	470	14.084	<0.0001	Impr	486	15.398	<0.0001	Impr	478	3.404	0.001	Impr	
CPAQ^	472	−14.237	<0.0001	Impr	492	−15.741	<0.0001	Impr	484	−3.633	0.000	Impr	
PHQ9	475	10.356	<0.0001	Impr	497	11.123	<0.0001	Impr	488	2.288	0.023	Impr	
GAD7	472	8.586	<0.0001	Impr	495	10.18	<0.0001	Impr	486	2.853	0.005	Impr	
NPOQ	462	5.048	<0.0001	Impr	475	7.56	<0.0001	Impr	468	3.223	0.001	Impr	
RMDQ	470	11.212	<0.0001	Impr	488	11.858	<0.0001	Impr	480	2.338	0.020	Impr	
TSK17	474	14.432	<0.0001	Impr	496	11.853	<0.0001	Impr	488	−0.422	0.673	Maint	
BPI severity	461	7.066	<0.0001	Impr	487	5.851	<0.0001	Impr	475	−0.267	0.7897	Maint	
BPI interference	458	10.585	<0.0001	Impr	483	11.086	<0.0001	Impr	471	1.863	0.0631	Maint	

**NB**: PMP – Pain management programme, PCS – Pain catastrophising scale, CPAQ – chronic pain acceptance questionnaire, PHQ-9 – patient health questionnaire, GAD-7 – generalized anxiety disorder assessment, NPO – negative problem orientation, RMDQ – Roland and Morrise disability questionnaire, TSK-17 – Tampa scale of kenesiophobia, BPI – brief pain inventory. * = *p* < 0.05, ** = *p* < 0.01, *** = *p* < 0.001. A higher score is better in all physical and psychological metrics except for those denoted with^.

**Table 8.** Bonferroni corrected post-hoc comparisons between each PMP visit for Intensive PMP. The direction of the significant change is noted as improved (Impr), maintained improvement (Maint) or worsened (Worse).

Outcome measure	Post hoc – intensive												
	Pre vs post				Pre vs 6 month post					Post vs 6 month post			
	DF	T-value	*p*-value	Effect	DF	T-value	*p*-value	Effect	DF		T-value	*p*-value	Effect
5 min Walk^	387	−25.946	<0.0001	Impr	406	−20.351	<0.0001	Impr	401		0.334	0.739	Maint
20 m Walk	390	11.252	<0.0001	Impr	420	7.891	<0.0001	Impr	413		−1.17	0.243	Maint
1 min Step-up^	391	−24.109	<0.0001	Impr	418	−17.823	<0.0001	Impr	412		1.536	0.125	Maint
PCS	433	18.058	<0.0001	Impr	453	15.254	<0.0001	Impr	446		−0.874	0.383	Maint
CPAQ^	431	−18.835	<0.0001	Impr	452	−16.009	<0.0001	Impr	443		0.802	0.423	Maint
PHQ9	433	20.993	<0.0001	Impr	452	13.459	<0.0001	Impr	445		−5.205	<0.0001	Worsen
GAD7	432	17.674	<0.0001	Impr	451	12.135	<0.0001	Impr	443		−3.581	<0.001	Worsen
NPOQ	426	9.947	<0.0001	Impr	441	10.389	<0.0001	Impr	434		1.563	0.119	Maint
RMDQ	433	20.018	<0.0001	Impr	454	13.444	<0.0001	Impr	446		−4.33	<0.0001	Worsen
TSK17	433	17.593	<0.0001	Impr	454	13.042	<0.0001	Impr	447		−2.619	0.009	Worsen
BPI severity	424	14.953	<0.0001	Impr	441	8.277	<0.0001	Impr	435		−4.871	<0.0001	Worsen
BPI interference	420	23.585	<0.0001	Impr	434	13.975	<0.0001	Impr	431		−7.075	<0.0001	Worsen

**NB**: PMP – Pain management programme, PCS – Pain catastrophising scale, CPAQ – chronic pain acceptance questionnaire, PHQ-9 – patient health questionnaire, GAD-7 – generalized anxiety disorder assessment, NPO – negative problem orientation, RMDQ – Roland and Morrise disability questionnaire, TSK-17 – Tampa scale of kenesiophobia, BPI – brief pain inventory. * = *p* < 0.05, ** = *p* < 0.01, *** = *p* < 0.001. A higher score is better in all physical and psychological metrics except for those denoted with ^.

• **PCS:**

*Standard PMP:* The mean pre-PMP PCS score was not classified as clinically relevant levels of catastrophising (PCS score >30/52). However, the score almost halved between pre-PMP and 6-month follow-up.
*Intensive PMP:* The mean pre-PMP PCS score was classified as clinically relevant (PCS score >30/52)^25^ and the score almost halved between pre-PMP and 6-month follow-up.

• **CPAQ:**

*Standard PMP:* The mean CPAQ score significantly increased (i.e. pain acceptance improved) from pre-PMP to post-PMP, and from post-PMP to 6-month follow-up.
*Intensive PMP:* The mean CPAQ score significantly increased from pre-PMP to post-PMP, and this score was maintained at 6-month follow-up.

• **PHQ-9**:

*Standard PMP:* The mean pre-PMP PHQ-9 score was classified as moderate depression (score = 10 > 15), whilst both post-PMP and 6-month follow-up scores fell into the mild depression category (score = 5 > 10).
*Intensive PMP:* The mean pre-PMP PHQ-9 score was classified as moderately severe (score = 15 <> 0). Post-PMP mean score saw a drop down two categories to mild depression (score = 5 > 10). The score at the 6-month follow-up slightly increased to within the moderate depression classification (score = 10 > 15).^30^.

• **GAD7:**

*Standard PMP:* The mean pre-PMP GAD7 score of the standard programme was at the high end of mild anxiety classification (score = 5 > 10). This score improved at 6-month follow-up and was close to being classified as not having anxiety (<5).
*Intensive PMP:* The mean pre-PMP GAD7 score was classified as moderate (score of 10–15) and dropped to mild (score = 5 > 10) at both post-PMP and 6-month follow-up.^22^.

• **NPO:**

*Standard PMP:* The standard PMP reduced NPO scores post-PMP and maintained this at 6-month follow-up.
*Intensive PMP:* The intensive PMP reduced NPO scores post-PMP and maintained this at 6-month follow-up.

• **RMDQ:**

*Standard PMP:* The mean pre-PMP RMDQ score reduced overall (pre vs 6-month post-PMP) by approximately 4 marks – a clinically important improvement is regarded to be ≥4 reduction.^31^
*Intensive PMP:* The mean pre-PMP RMDQ score reduced 6.5 between pre- and post-PMP. The score worsened at 6-month follow-up. However, the overall (pre vs 6-month follow-up) change remained a clinically important improvement (≥4).^31^.

• **TSK-17** 

*Standard PMP:* The mean pre-PMP score of TSK-17 was 1-point below being considered a high level of kinesiophobia (>37). The score improved across both subsequent timepoints.
*Intensive PMP:* The mean pre-PMP was classified as a high level of kinesiophobia. The score improved at post-PMP and was no longer classified as high kinesiophobia. Despite a significant worsening of the score at 6-month follow-up, the score remained outside of the high category.^24^.

• **BPI – severity and interference**

*Standard PMP:* From pre-PMP to post-PMP a clinically meaningful change (min 10% reduction^13^) was seen in pain severity. This score was maintained to 6-month follow-up.
*Intensive PMP:* BPI-severity and BPI-interference significantly worsened at the 6-month follow up compared to the post-PMP score. A clinically meaningful increase in pain severity was seen at 6-months post-intensive PMP, that is, over 10% increase in severity.

## Discussion

This service evaluation aimed to (1) evaluate the screening process of chronic pain patients for either a standard or intensive PMP delivered at Manchester and Salford Pain Centre, and (2) determine the effectiveness of the two PMPs. Gender distribution across the PMPs was similar, with women making up around 70% of participants on each PMP, reflective of the UK prevalence of chronic pain.^3^ Those on the standard PMP were more likely to be in paid employment, whereas a greater proportion of intensive PMP attendees were unemployed, often because their pain prevented them from being able to work. In terms of diagnosis, a greater proportion of people with chronic widespread pain, fibromyalgia and osteoarthritis were referred to the intensive PMP, perhaps due to the increasing psychological distress and disability caused by such pain presentations.^33^ Individuals with neck and arm pain were more commonly referred to the standard PMP. On all physical and psychological outcomes, those referred to the intensive PMP demonstrated greater psychological distress, lower pain acceptance and poorer physical function than those referred to the standard PMP. This demonstrates that the screening process at MSPC can identify individuals with greater pain-related distress and disability, and ensure that they receive appropriate intensive intervention.

This service evaluation also aimed to explore the effectiveness of each PMP individually, including longer-term follow-up to 6-months post-PMP. The standard PMP demonstrated significant improvements in all outcomes from baseline to post-course, and from baseline to 6-month follow-up, with pain catastrophizing, pain acceptance, anxiety, rumination and negative problem orientation continuing to improve significantly after the course had ended. The intensive PMP demonstrated significant improvements in all outcomes from baseline to post-course, and from baseline to 6-month follow-up. In particular, depression improved to a clinically meaningful degree across timepoints in the intensive PMP, and disability showed a minimum 30% improvement at post-PMP and 6-months post-PMP. Likewise in both PMPs, a clinically meaningful (10% minimum) reduction was seen in pain severity from pre- to post-PMP.

Despite the improvements seen at the end of both PMPs, some key outcomes started to deteriorate to 6-month follow-up within the intensive PMP, including depression severity, disability, pain severity and pain interference. For pain severity, the subsequent increase was clinically meaningful (i.e. increased by more than 10%) and therefore has potential to impact on patients’ lives. The positive change seen in the intensive PMP is therefore not maintained to the same degree as the standard PMP for these outcomes but still remains better than at baseline. Importantly, depression and disability did not worsen to a clinically meaningful degree between post-PMP and 6-month follow-up. Therefore the changes in these outcomes (including the increase in pain severity) may be less impactful on one’s life than is alluded to by statistical outcomes.

This is an important finding, particularly for the outcome of pain severity, with implications for the psychological and physical wellbeing of intensive PMP attendees, particularly longer term if any outcomes continue to deteriorate. This finding may arise due to the nature of the intensive PMP being a 3-week full-time programme with more time spent developing management skills and receiving support from the PMP team and implementing learning in the protective environment of the pain centre. It may be that once the PMP and its associated intensive support ends, there is a greater risk of bounce back towards previous levels of pain and depressive symptoms when faced with challenges upon leaving the PMP, particularly as patients have less experience than those on the standard PMP in putting their learning into practice in everyday life. This contrasts with other work suggesting that intensive PMPs should demonstrate greater and more sustained gain,^5^ and suggests a need for further work to establish the mechanisms underlying sustained gain in order that these may be promoted to protect against the deterioration seen in this evaluation. For example, future work might examine the impact of providing 75 hours of contact time (as done in the intensive PMP) across two days per week, across 7–8 weeks in a manner similar to the standard PMP, to examine the extent to which the three-week, full-time nature of the intensive PMP contributes to the deterioration in outcomes seen in the present evaluation. Alternatively, given the groups undertaking the standard vs intensive PMPs differed significantly in physical capability and psychological distress, future work might compare the PMPs with groups with similar baseline measures to more confidently establish effectiveness of the PMPs, and for whom they are most effective.

The deterioration seen may also be compounded by the complex interlinking of chronic pain and depression (found across a variety of pain conditions^33^), each of which can exacerbate the other, along with a host of other physical and psychological outcomes. Those on the intensive PMPs demonstrate significantly greater distress and pain severity and interference, which evidence suggests may lead to longer duration of symptoms.^34^ Indeed, self-reported pain severity is likely influenced by mood (lower mood predicting greater pain severity^35^), and for those in greater distress these outcomes are more strongly associated with one another and therefore more difficult to disentangle.^33^ Such intensity and complexity of symptoms may not be sufficiently addressed in an intensive PMP with little opportunity to implement learning in everyday life. This may warrant the use of further ‘booster’ sessions to ‘top up’ the PMP learning and provide important support and feedback to attendees as they learn to implement their new pain management skills in their usual daily life.

In contrast with the recent NICE guidance^7^ on PMPs, this evaluation presents evidence of the beneficial (statistically and clinically) effects of both standard and intensive PMPs for the improvement of physical and psychological outcomes for a sample of people with diverse chronic pain conditions. This evaluation demonstrates the usefulness of such PMPs and outlines areas for further work to address the maintenance of positive change post-PMP attendance. Early identification of PMP attendees at risk of either not responding or deteriorating after completion of a PMP may be an important next step in ensuring that both established and emerging PMPs are tailored to address the specific needs of those most at risk of physical and psychological distress. Attendees within this cohort have gone through a robust screening and clinical assessment process prior to the PMP and this information considers barriers to PMP engagement, directing potential candidates to alternative interventions within the service. The follow-up sessions at 1, 3 and 6-months post PMP allows clinicians to identify participants who may have deteriorated but beyond 6 months, this relies on patients proactively contacting the service highlighting the requirement for a more robust 12-month follow-up process. This will also be important in establishing/reinforcing safeguards against adverse effects/deterioration post-PMP, especially for the outcome of pain severity which, if left unmanaged, may consequently negatively affect mood and other important pain-related outcomes.

In summary, this service evaluation has found that the two PMPs studied demonstrate benefit in terms of physical and psychological outcomes, with some outcomes, in particular pain intensity, deteriorating at longer-term follow-up. The findings indicate a need to examine the mechanisms of PMPs and ensuring that attendees have opportunities to practice their skills development in everyday life whilst engaging with a PMP, especially for those on more intensive or residential programmes, to minimise risks of deterioration post-completion.

## Limitations and future research

A key strength of the current paper is its presentation of the results of two PMPs that are well established and running in practice, with analysis of real-world data including longer-term 6-month follow-up of outcomes. This means that the results presented are more representative of outcomes in practice as opposed to findings from controlled studies where inclusion criteria may be more stringent and exclusive of individuals showing greater psychological distress. However, this also means that the current evaluation lacked the methodological control that would be present in a randomized controlled trial (e.g.), and that causality, whilst possible, cannot be inferred.

Another limitation of the present work is the lack of longer term follow up data. Although MSPC request PMP completers to provide 12-month follow-up data, response rates remain low, and with little indication of the reasoning for not completing the follow-up assessments. For example, it may be that PMP completers do not respond to 12-month follow-ups because their symptoms have maintained/improved and they do not feel a need to report back, or because their symptoms have worsened and this prevents responding. In practice, improving methods of obtaining longer term follow-up data will benefit future analysis to examine how outcomes change over a longer period, and the extent to which the outcomes identified in this evaluation as worsening in the intensive PMP continue to worsen or whether these stabilize. This would then have implications for the need for further service delivery in the form of ‘booster’ sessions. Given the diversity within PMP attendees in terms of demographics, future work would also benefit from assessing for whom the PMP works best for through subgroup analysis. In the context of the present evaluation, it would be beneficial to examine what demographic and baseline clinical outcomes are influential in the maintenance or deterioration of outcomes post-PMP to establish whether further tailoring of PMPs is required for certain groups to maximise outcomes.
